# Platelet Integrins in Tumor Metastasis: Do They Represent a Therapeutic Target?

**DOI:** 10.3390/cancers9100133

**Published:** 2017-09-28

**Authors:** Marion Lavergne, Emily Janus-Bell, Mathieu Schaff, Christian Gachet, Pierre H. Mangin

**Affiliations:** Université de Strasbourg, INSERM, EFS Grand-Est, BPPS UMR-S 949, FMTS, F-67000 Strasbourg, France; marion.lavergne@efs.sante.fr (M.L.); Emily.Janus-Bell@efs.sante.fr (E.J.-B.); mathieu-schaff@outlook.com (M.S.); christian.gachet@efs.sante.fr (C.G.)

**Keywords:** platelets, cancer, integrins, hemostasis, thrombosis, metastasis, antiplatelet agents

## Abstract

Platelets are small anucleated cell fragments that ensure the arrest of bleeding after a vessel wall injury. They are also involved in non-hemostatic function such as development, immunity, inflammation, and in the hematogeneous phase of metastasis. While the role of platelets in tumor metastasis has been recognized for 60 years, the molecular mechanism underlying this process remains largely unclear. Platelets physically and functionally interact with various tumor cells through surface receptors including integrins. Platelets express five integrins at their surface, namely α2β1, α5β1, α6β1, αvβ3, and αIIbβ3, which bind preferentially to collagen, fibronectin, laminin, vitronectin, and fibrinogen, respectively. The main role of platelet integrins is to ensure platelet adhesion and aggregation at sites of vascular injury. Two of these, α6β1 and αIIbβ3, were proposed to participate in platelet–tumor cell interaction and in tumor metastasis. It has also been reported that pharmacological agents targeting both integrins efficiently reduce experimental metastasis, suggesting that platelet integrins may represent new anti-metastatic targets. This review focuses on the role of platelet integrins in tumor metastasis and discusses whether these receptors may represent new potential targets for novel anti-metastatic approaches.

## 1. The Role of Platelets in Hemostasis and Tumor Metastasis

Blood platelets are small anucleate cell fragments derived from megakaryocytes. They are major actors in hemostasis, which represents the physiological process preventing spontaneous bleeding and leading to the arrest of blood loss in case of vascular injury [1]. Following vessel injury, platelets adhere to various adhesive proteins exposed by the subendothelium. Under conditions of elevated blood flow, found notably in the microcirculation, platelet recruitment is primarily ensured by the glycoprotein (GP) Ib-IX-V complex, which supports binding to von Willebrand factor (VWF) immobilized in the subendothelium [2]. Integrins have a similar function, allowing the capture of flowing platelets, but this is restricted to low wall shear rate conditions (<1000 s^−1^) [3,4]. Integrins support stable adhesion of platelets and also initiate their activation through outside-in signaling. Platelets express five integrins, namely αIIbβ3, αvβ3, α2β1, α5β1, and α6β1, which bind preferentially fibrinogen, vitronectin, collagen, fibronectin, and laminins, respectively [5]. Stationary platelet adhesion facilitates the interaction of glycoprotein (GP)VI with collagen [6], which initiates an intracellular signaling cascade, leading notably to the release of the δ-granules content including adenosine di-phosphate (ADP), adenosine tri-phosphate (ATP) [7], and serotonin, and to the synthesis of thromboxane A2 (TxA2). These soluble agonists, together with thrombin, which is generated at the site of injury, potentiate platelet activation, resulting in the upregulation of the affinity of integrin αIIbβ3 for its main ligand, soluble fibrinogen [8]. Fibrinogen forms bridges between adjacent platelets, supporting the formation of a plug that seals the breach and stops blood loss [9]. Besides their main role in hemostasis, platelets were proposed to participate in non-hemostatic functions, including development, wound healing, inflammation, angiogenesis, and cancer [10,11,12,13,14].

In 1865, Armand Trousseau clinically recognized the link between cancer and hemostatic abnormalities. He reported cases of thrombophlebitis in patients who were diagnosed with cancer [15]. In 1968, Gasic and collaborators reported the first link between platelets and tumor metastasis. They demonstrated that the ability of inoculated tumor cells to colonize the lung was markedly decreased in thrombocytopenic mice [16]. The link with platelets was even further evidenced after transfusion of platelets which restored metastasis in the thrombocytopenic mice. Besides experimental work, recent clinical studies have also proposed that platelets might participate in tumor metastasis. This mainly results from a meta-analysis of large clinical trials on patients with cardiovascular diseases that highlighted the beneficial effect of the anti-platelet drug aspirin being taken daily, which reduced the incidence of metastasis in adenocarcinomas (stomach, small bowel, pancreas, bileduct, colon, rectum, uterus, ovary, and prostate cancer) and breast cancer [17,18] and notably reduced deaths due to colorectal and gastrointestinal cancers [19].

**Figure 1:**
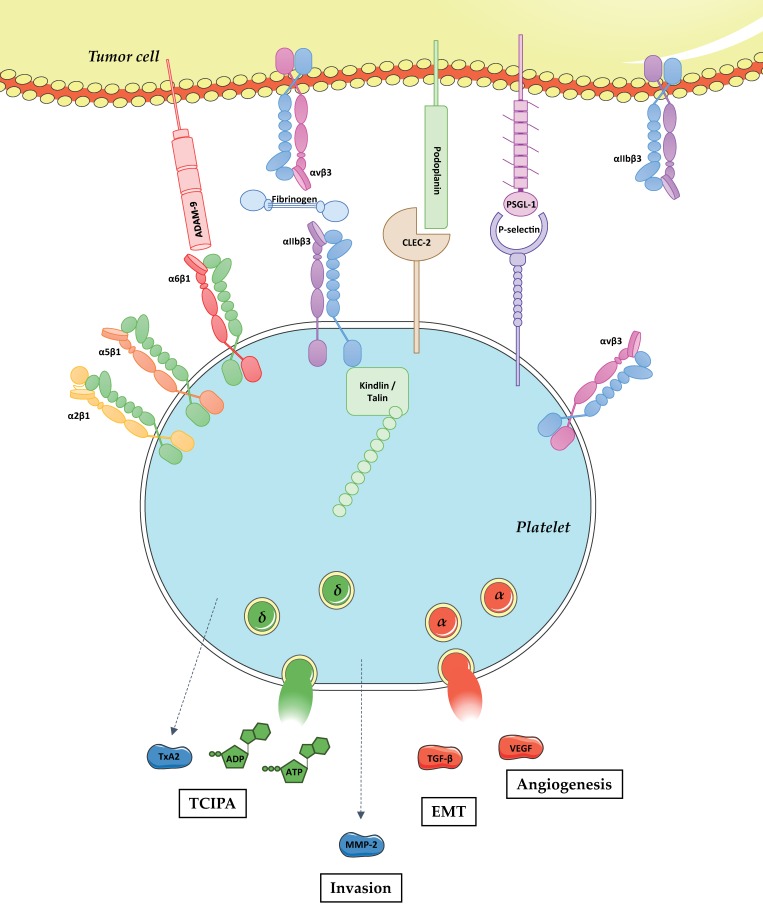
**Physical and functional platelet–tumor cell crosstalk.** Tumor metastasis is a complex process including the detachment of tumor cells from the primary tumor, intravasation, survival in the bloodstream, extravasation, and proliferation at the distant site. Following intravasation, tumor cells enter into the bloodstream and get in close contact with various circulating blood cells including platelets. Platelets physically interact with tumor cells through the binding of CLEC-2, P-selectin, and integrins α6β1 and αIIbβ3 with podoplanin, PSGL-1, ADAM-9 and fibrinogen/αvβ3, respectively. The role of platelet integrins α2β1, α5β1, and αvβ3 in direct interaction with tumor cells remains unknown. Platelet adhesion to tumor cells results in their activation, which promotes: (i) platelet shape change; (ii) integrin αIIbβ3 activation upon talin and kindlin binding to the intracytoplasmic domain of the β3 chain; (iii) the release of biologically active molecules including TxA2, ADP, ATP, MMP-2, TGF-β, and Vascular endothelial growth factor (VEGF). In turn, these mediators promote: (i) Tumor cell induced platelet aggregation (TCIPA); (ii) tumor cell invasion; (iii) EMT; and (iv) angiogenesis.

Numerous studies have tried to define the role of platelets in tumor metastasis. Platelets are likely to be among the first blood cells to interact with tumor cells upon intravasation. It has been reported that the platelet receptors C-type lectin-like receptor 2 (CLEC-2) [20], P-selectin [21], and integrins α6β1 and αIIbβ3 [22,23] support interaction with tumor cell through the binding of podoplanin, P-selectin glycoprotein ligand-1 (PSGL-1), A disintegrin and metalloproteinase domain-containing protein 9 (ADAM-9), and fibrinogen/αvβ3, respectively. This interaction was proposed to form a physical shield around cancer cells, thereby protecting them from the deleterious effects of shear forces [24]. Platelets have also been proposed to protect the tumor cells from the immune system [25]. This observation was brought to light in a study showing that platelets contribute to metastasis by protecting tumor cells from natural killer (NK) cell lysis [26]. The proposed mechanism could rely on the ability of platelets to secrete agents such as transforming growth factor-β (TGF-β) which down-regulates the expression of natural killer group 2 member D (NKG2D) on NK cells, decreasing their cytotoxic effect [27]. An additional mechanism could result from the ability of platelets to transfer a major histocompatibility complex (MHC) class I onto tumor cells to provide a self-signal to NK cells which will suppress their cytotoxic activity on these tumor cells [25]. Beside these protective roles, platelets promote epithelial-mesenchymal transition (EMT), notably by the ability of tumor cell activated platelets to release TGF-β [28]. Moreover, it has been shown that platelets facilitate tumor cell adhesion to endothelial cells through a mechanism that could rely on αIIbβ3 [29]. Finally, platelets were proposed to play a role in the enhancement of tumor cell extravasation accross the endothelial barrier. This has been reported in a study showing that tumor cell transendothelial migration is allowed thanks to tumor cell-activated platelets release of ATP which induces endothelial barrier opening upon binding the endothelial P2Y_2_ (P2Y_2_) receptor [30]. Platelets could also facilitate tumor cell extravasation after the release of MMPs, which degrades the extracellular matrix. This effect could be direct after the release of matrix metalloproteinase-2 (MMP-2) by activated platelets [31], or indirect through the ability of platelets to stimulate the secretion of MMPs by tumor cells [32] (Figure 1).

While the role of platelets in tumor metastasis has been long recognized and extensively reviewed [13,14,33,34,35], the underlying molecular mechanism remains largely unknown. This review focuses on the current knowledge about the potential role of platelet integrins in tumor metastasis. We will first provide some general information about integrins expressed on platelets and highlight their main known functions. We will also summarize the experimental evidence that has been reported concerning their involvement in physical and functional interaction with tumor cells and describe their proposed role in experimental tumor metastasis. Finally, we will discuss whether platelet integrins could represent a novel and interesting anti-metastatic target.

## 2. The Repertoire and Function of Integrins at the Platelet Surface

Platelets express five distinct types of integrins at their surface which belong either to the β1 or the β3 family. The best-known function of platelet integrins is to ensure the adhesive properties of platelets through their interaction with plasma or subendothelial proteins [36]. Integrins oscillate between various structural conformations going from a low to more elevated affinity state for their ligands [37,38]. In resting platelets, integrins are recognized to be in a relatively low-affinity state for their ligands and are unable to efficiently bind them in suspension. However, when those ligands including fibrinogen, collagen, fibronectin, or laminins are immobilized on a surface, platelet integrins support the recruitment of resting discoid platelets under low blood flow conditions (<1000 s^−1^). This observation indicates that even in resting platelets, at least one pool of surface expressed integrins has a sufficient activation status allowing them to bind their ligands [2,3]. To move from a low to a high affinity state, integrins become activated by a so-called inside-out signaling arising from stimulation by soluble mediators (ADP, TxA2, thrombin) or adhesive proteins (collagen, VWF) [39,40]. These signaling cascades activate heterotrimeric G proteins or tyrosine kinases leading to phospholipase C (PLC)-β or -γ activation, increase in intracellular levels of Ca^2+^ and lead to the activation of Ca^2+^ and diacylglycerol-regulated guanine-nucleotide-exchange factor I (CalDAG-GEFI) and Rap1b [41]. This results, at least for αIIbβ3, is the binding of talin-1 and kindlin-3 to the cytoplasmic tail of the β chain, triggering a conformational change in the extracellular domain resulting in a shift from a low to a high affinity state of the integrin for its ligands [42]. In turn, ligand binding to the integrins generates an outside-in signal that mainly reinforce platelet activation (see below).

### 2.1. The Platelet β1 Integrins

Members of the β1 integrin family are ubiquitously expressed and found notably on lymphocytes, epithelial, endothelial and smooth muscle cells (SMCs). Integrins β1 are involved in hallmarks of cancer and particularly in survival, cell death control, and invasion of tumor cells [43].

Platelets express three members of this family of proteins, namely α2β1, α5β1, and α6β1, which bind collagen, fibronectin, and laminins, respectively. These integrins are expressed at relatively low levels on the platelet surface reaching a couple of thousands molecules per cell [5]. Even though no precise quantification has been reported, α6β1 has probably the highest copy number per platelet compared to the other two β1 integrins [44].

#### 2.1.1. α2β1

Integrin α2β1 is expressed by platelets, fibroblasts, epithelial, and endothelial cells [45]. It senses many different extracellular matrix proteins including collagen and laminins. This integrin plays a role in various processes including immunity, development, and cancer [46]. *Integrin α2* gene polymorphisms were shown to be associated with breast and colorectal cancer risk [47,48,49].

On platelets, integrin α2β1 is a receptor for collagen which mainly supports the stable adhesion of platelets [50]. Because of a polymorphism, its surface expression varies between 2000 and 8000 copies per platelet [51]. Ligand binding to α2β1 generates outside-in signals involving Src kinases, Syk, the adapter protein SLP-76 and leading to PLC-γ2 activation and subsequent mobilization of internal stores of Ca^2+^ [52]. Integrin α2β1 does not appear to play a crucial role in hemostasis. Indeed, two patients with genetic defects in α2β1 exhibit only a moderate bleeding phenotype [53,54]. These results are in agreement with a mouse model deficient for α2β1 that did not show a prolonged tail-bleeding time [55]. The use of knock-out mice helped to identify a role for integrin α2β1 in experimental thrombosis. Interestingly, this integrin appears to be involved at the blood-vessel wall interface as well as in the process of thrombus stability [56,57]. Studies performed in our laboratory did not conclude that there was a major role of α2β1 in two models of experimental thrombosis based on mechanical injury of the aorta and FeCl_3_ injury of the carotid artery, suggesting that this integrin most likely plays a subtle role in experimental thrombosis (Mangin, unpublished results, 2015). Polymorphisms shown to increase the expression level of α2β1 and platelet adhesiveness were also proposed as risk factors for thrombotic events [58]. No other role for platelet α2β1 has been reported to date.

#### 2.1.2. α5β1

Integrin α5β1 is ubiquitously expressed and best known as a receptor for fibronectin, playing an important role in cell migration and differentiation especially during development [59]. For this reason, α5 deletion in mice is lethal at the embryonic stage, which has precluded the investigation of its function in various cells including platelets [60]. Integrin α5β1 is overexpressed in several cancers, including colon, breast, ovarian, lung, and brain tumors, and is associated with a poor prognosis [61]. It has been proposed that targeting α5β1 expressed on tumor cells might reduce the metastasis of head and neck cancers [62]. Clinical trials on the anti-α5β1 chimeric antibody, M200 (Volociximab), have shown it to be generally well tolerated, with some preliminary evidence of efficacy in advanced non-small-cell lung cancer [63].

Concerning platelets, it has been reported that α5β1 supports modest platelet adhesion and activation to immobilized soluble fibronectin under both static and flow conditions [64,65,66]. We have previously shown that α5β1-mediated platelet adhesion and activation becomes much more significant when platelets are perfused over cellular fibronectin in its fibrillar form, as found in the vessel wall [67]. The role of platelet α5β1 in hemostasis, arterial thrombosis, and beyond remains to be established.

#### 2.1.3. α6β1

Integrin α6β1 is a ubiquitous receptor for laminins that is notably known to structure epitheliums [68]. Patients with a deficiency in the *α6* gene suffer from a painful disease called epidermolysis bullosa [69], which is a rare genetic connective tissue disorder characterized by blistering of the skin [70]. α6β1 has also been reported to be overexpressed in breast and prostate cancer and in glioblastoma [71,72,73]. It was proposed to favor tumor cell survival as well as tumor metastasis [74,75].

Concerning platelets, several studies have shown that α6β1 supports platelet adhesion to laminins under both static and flow conditions [76,77,78,79,80]. This interaction generates outside-in signals involving the tyrosine kinase Syk, PLC-γ2, Phosphoinositide 3-kinase (PI3K), and Cdc42, leading to morphological changes in platelets [77,81,82]. By using tissue-specific α6β1-deficient mice we showed that this integrin supports platelet adhesion to vascular laminins even under high wall shear rate conditions. Moreover, α6β1 is not essential for normal hemostasis but plays an important role in experimental thrombosis [44]. We have also established a role for this platelet integrin in tumor metastasis (see Section 3.1).

### 2.2. The Platelet β3 Integrins

αvβ3 and αIIbβ3 are the two members of the β3 integrin family. αvβ3 is expressed on osteoclasts and endothelial cells, while αIIbβ3 is mainly found in platelets [83]. Both integrins have been described to be expressed in tumor cells [22,84,85,86]. While the role of αIIbβ3 in tumor cells remains unclear, the importance of αvβ3 has been much better defined. The expression level of αvβ3 integrin is correlated to a metastatic phenotype in cervical, ovarian, pancreatic, prostate, and breast cancer, glioblastoma, and melanoma [87]. αvβ3 is involved in many steps of the tumor progression as well as in endothelial cell survival to allow angiogenesis [88], migration, and metastasis [89,90].

#### 2.2.1. αIIbβ3

Integrin αIIbβ3 was long believed to be specifically expressed in the platelet lineage, but has also been reported to be present in several tumor cells [22,84,85,86], promoting tumor cell adhesion and invasion [22,85,86,91]. Integrin αIIbβ3 is the most abundant receptor on platelets, with 50,000 copies at the surface and 30,000 more in the open canalicular system and α-granules that are exposed upon platelet activation [92]. αIIbβ3 notably recognizes RGD peptide-binding sequence on various adhesive proteins including fibrinogen, VWF, fibronectin, and vitronectin. The major role of αIIbβ3 is to ensure platelet aggregation through the binding of plasma fibrinogen, whose dimeric nature allows the bridging of adjacent platelets [36]. This process requires αIIbβ3 to be in an activated state. The physiological importance of αIIbβ3 is evidenced by a bleeding diathesis called Glanzmann’s thrombosthenia, which results from a deficiency of the integrin and is characterized by a defect in platelet aggregation [93]. αIIbβ3 is also a target for a class of anti-thrombotic drugs used in acute settings such as during myocardial infarction and percutaneous coronary interventions [94].

Fibrinogen binding to αIIbβ3 induces its clustering and initiates outside-in signaling that has been extensively studied. This signaling cascade was proposed to involve Src kinases, Syk, SLP-76, PI3K p110-β and -δ, and leads to the activation of PLC-γ2 and Rap1b [95,96]. Outside-in signaling reinforces the signal leading to αIIbβ3 activation, and is also responsible for morphological changes of platelets, granule secretion and clot retraction. It plays an important role in the stabilization of aggregates and is central in both hemostasis and experimental thrombosis [97,98,99,100,101].

#### 2.2.2. αvβ3

Integrin αvβ3 is mainly found in endothelial cells, SMCs, and platelets [59]. Its structure is closely related to that of αIIbβ3 and recognizes RGD peptide-binding sequence in several adhesive proteins including fibrinogen, fibronectin, VWF, and vitronectin. Only several hundred copies of αvβ3 are found at the platelet surface. It has been proposed that αvβ3 supports modest platelet adhesion to both fibronectin and vitronectin [66,102] and might participate in clot retraction [103]. Its importance in hemostasis and arterial thrombosis has not yet been reported. Recent unpublished results from our group indicate that αvβ3 plays a minor role in both processes as evidenced by a normal tail-bleeding time and no impact on experimental thrombosis in a mouse strain knocked out for this integrin in the platelet lineage (PF4-Cre-αv^−/−^), (Pierre Mangin, Catherine Léon, unpublished results, 2016).

## 3. The Role of Platelet Integrins in the Interplay with Tumor Cells and in Tumor Metastasis

### 3.1. The β1 Integrins

Based on both experimental and spontaneous models, we have recently reported that platelet integrin α6β1 supports tumor metastasis [23]. The role of this integrin appears to be linked to its ability to directly interact with various types of tumor cells through the binding of ADAM-9 [104]. We provided evidence that this interaction is important in the process of tumor cell extravasation. We hypothesize that the decreased physical interaction of platelets with tumor cells reduces the number of agents released by platelets, including secreted ATP, which is known to facilitate extravasation through acting on endothelial P2Y_2_ [30]. Whether the other members of the β1 integrin family, α2β1, and α5β1, also participate in metastasis is still unknown. We could speculate that integrins probably do not play a crucial role in this process, based on the observation that the level of inhibition of tumor cell colonization to the lungs was very similar in mice deficient for platelet α6β1 when compared to mice deficient for all three β1 integrins (PF4-Cre-β1^−/−^) [23]. However, experimental evidence is needed to precisely assess the potential role of platelet α2β1 and α5β1 in tumor metastasis.

### 3.2. The β3 Integrins

Seminal studies have shown that β3 antagonists such as blocking antibodies or RGD-containing peptides inhibit the physical interaction between platelets and tumor cells, allowing the authors to suggest that platelet αIIbβ3 ensures the direct binding of platelets with tumor cells [22,105,106,107]. However, because αIIbβ3 was also reported to be expressed in tumor cells [22,84,85,86] and has even been proposed to mediate the direct interaction with platelets [108], the implication and the relative importance of platelet versus tumor cell αIIbβ3 in the physical interaction of these cells long remained unclear [91,109]. Since then, the use of platelets from Glanzmann’s thrombasthenic patients, who lack functional αIIbβ3, allowed confirmation of the role of this integrin in direct platelet/tumor cell interaction in a static adhesion assay [22,29]. Flow-based assays indicated that αIIbβ3 facilitates the stable adhesion of tumor cells on immobilized platelets, a function that is well known to be ensured by this integrin on various adhesive proteins [110]. Additional studies showed that platelet αIIbβ3 supports interaction with tumor cells through αvβ3 in a process relying on fibrinogen, which could bridge both integrins [29,111,112,113]. While it is clear that tumor cell binding to platelets promotes activation, as evidenced by shape change, granule content secretion, or TxA2 release [34,114,115,116,117,118,119,120,121,122,123], it is rather challenging to evaluate the importance of αIIbβ3 in this process relative to other platelet receptors binding to tumor cells. This is even more challenging if we assume that the physical links between platelets and tumor cells depends on the repertoire of receptors expressed on different tumor cells. Nevertheless, Amirkhosravi and colleagues proposed that tumor cell binding to platelet αIIbβ3 promotes their activation and induces the release of VEGF, which can act on tumor cells to regulate their function [124].

Platelet integrin αIIbβ3 has been reported to play a central role in the process of TCIPA [125,126,127,128]. This observation is not a surprise because αIIbβ3 is a key receptor supporting platelet aggregation [36]. While the primum movens of TCIPA remains elusive, one could speculate that platelet αIIbβ3 participates at least partially because of its ability to ensure the physical interaction with tumor cells [127,129]. The process of TCIPA has been extensively studied and well characterized. In addition to αIIbβ3, it is supported by other platelet surface receptors such as the GPIb-IX-V complex and CLEC-2 and triggered by soluble agonists including ADP and TxA2, which are released from activated platelets [20,122,126,130,131,132,133]. TCIPA also relies on thrombin generation, even though the role of this serine protease largely depends on the experimental conditions [115,132,134,135,136,137]. Several studies have shown a correlation between TCIPA and tumor metastasis [85,118,128,138,139,140,141,142,143,144]. This is now widely accepted since many publications refer to this link, even though the detailed analysis of the seminal papers mainly show indirect links, with a paucity of experimental evidence (Figure 1).

It has been convincingly reported that αIIbβ3 blockers impair experimental metastasis [145,146,147]. However, whether these inhibitors mediate their effect by acting on platelet or tumor cell αIIbβ3 integrins has long been unclear. Moreover, several αIIbβ3 blockers are not specific [148,149] and also target αvβ3, which is expressed in many tumor cells and well known to participate in cancer cell function and metastasis [87]. One of the most convincing studies supporting a role of platelet αIIbβ3 in metastasis came from Bakewell and collaborators, who showed that transfer of β3^−/−^ bone marrow in irradiated wild-type mice confers protection towards osteolytic metastasis [146]. However, this result has recently been challenged using αIIb-deficient mice [150]. While the authors observed a marked reduction of tumor cell accumulation into the lungs of αIIb^−/−^ mice several hours post-inoculation with the presence of smaller tumor cell clusters, tumor progression at late stages was markedly increased as compared to the wild type. This observation, which will require confirmation, also challenges the link between TCIPA and tumor metastasis.

The role of platelet αvβ3 in the physical and functional interaction with tumor cells as well as in experimental metastasis remains largely unclear. Given the very low number of copies of αvβ3 on the platelet surface, it is unlikely that this integrin plays a major role in this process, but a definitive answer cannot be reached without experimental evidence.

## 4. Is Targeting Platelet Integrins a Potentially Promising Anti-Metastatic Strategy?

Concerning β1 integrins, we have reported that platelet α6β1, but probably not α2β1 and α5β1, participates in tumor metastasis [23]. Moreover, we provided evidence that a pharmacological approach based on blockade of α6β1 with GoH3, an antibody, reduced experimental metastasis, suggesting that this integrin represents an interesting target for anti-metastatic therapy. Importantly, administration of the blocking antibody did not induce thrombocytopenia and did not impair hemostasis, suggesting that such an approach might not be associated with an elevated bleeding risk. However, because α6β1 is not just expressed on platelets, preclinical studies appear mandatory to evaluate the long-term impact of an anti-α6β1 agent. On the one hand, one could speculate that such treatment might be beneficial and could further reduce metastasis since α6β1 is notably expressed by endothelial and tumor cells, in which it participates in cancer progression [74,75]. On the other hand, targeting α6 might have deleterious effects because this integrin is ubiquitously expressed and plays various roles, notably in epithelial cell anchoring. As an example, a lack of α6 expression has been shown to result in hemidesmosome deficiency and be responsible for skin and mucous membrane disorders, including pyloric atresia and epidermolysis bullosa [69]. Whether a pharmacological approach in patients could induce such pathologies linked to development is uncertain but will clearly need to be evaluated in preclinical and clinical studies.

It is recognized that integrin αIIbβ3 plays a central role in both TCIPA and tumor metastasis and that there is a potential link between both processes [151]. It has also been shown that various αIIbβ3 blockers efficiently reduce experimental metastasis, allowing numerous groups to speculate that this integrin represents an attractive target to limit tumor metastasis [22,145,147,152]. αIIbβ3 blockers, including Abciximab, Eptifibatide, and Tirofiban, are already in clinical use as potent anti-thrombotic drugs [153] (Table 1). These agents are administered intravenously and their use is strictly restricted to acute settings such as percutaneous coronary interventions and acute coronary syndromes (ACS) because of their elevated risk of bleeding. It is therefore not conceivable to use such agents in the long term to prevent tumor metastasis. There have been attempts to develop orally active αIIbβ3 blockers, based on an RGD peptide-binding sequence. Unfortunately, the clinical trial was stopped before completion because of a non-significant benefit for patients with an ACS and an increase in mortality [154]. The underlying mechanism appears to be linked to the ability of these antagonists to activate αIIbβ3 through fibrinogen mimetic action on the ligand-induced binding site (LIBS) [155]. As a consequence, the development of new oral anti-αIIbβ3 agents was stopped. While the interest in developing novel anti-αIIbβ3 dropped, it has been postulated that the strategy rather than the target is inappropriate [156,157,158]. Contrary to anti-αIIbβ3, the use of agents targeting only the activated form of αIIbβ3 could be used at much lower systemic concentrations since they specifically accumulate at sites of platelet activation, therefore improving their safety profile. Among these new strategies (Table 1), one consists of targeting the active form of αIIbβ3 with a single-chain antibody (scFvMA2) that exclusively recognizes the active conformation of αIIbβ3 [158]. Another innovative agent, RUC-4, is a small molecule identified through high-throughput screening that targets the metal ion-dependent adhesion site (MIDAS) site of β3, presenting the advantage of preventing the high-affinity ligand-binding conformation change of the integrin [159]. Finally, a more recent strategy aims to target the plexin-semaphorin-integrin (PSI) involved in the Protein Disulfide Isomerase (PDI)-like activity of β3 integrin, which inhibits PDI-like activity and fibrinogen binding [160]. Whether these innovative agents exhibit a protective effect in mouse models of experimental metastasis is not yet known and will need to be investigated.

## 5. Conclusions

Platelet integrins, mainly α6β1 and αIIbβ3, have been shown to participate in the hematogeneous phase of metastasis. The main known role of these integrins is to support the physical interaction between platelets and tumor cells and to regulate their function. The relative importance of these glycoproteins compared to other platelet receptors involved in metastasis is still unclear. Experimental evidence has been provided that targeting these receptors efficiently reduces tumor cell colonization into the lungs, suggesting that they could represent interesting targets for anti-metastatic drugs. A clear drawback of targeting α6 is that this integrin is ubiquitously expressed and a pharmacological approach could have unwanted side effects. This will need to be evaluated in pre-clinical and clinical studies. The main drawback for αIIbβ3 is its key role in hemostasis. While acute treatment can be used in the setting of arterial thrombosis, it is impossible to consider long-term treatment with current clinically used anti-αIIbβ3 drugs. Future development of more specific agents targeting only activated forms of this integrin, which have a minimal effect on hemostasis, might be interesting but will first need to demonstrate protection towards metastasis in animal models.

## Figures and Tables

**Table 1:** Anti-αIIbβ3 agents.

Name	Nature of the Agent	Use	Inhibition of Platelet Aggregation	Inhibition of in Vivo Thrombus Formation	Activatory Effect on αIIbβ3	Effect on Bleeding
**Abciximab** **(ReoPro^®^)**	chimeric Fab fragment derived from the murine monoclonal antibody 7E3	Clinically used	√	√	√	√
**Tirofiban** **(Aggrastat^®^)**	non-peptide agent based on the RGD sequence	Clinically used	√	√	√	√
**Eptifibatide** **(Integrilin^®^)**	KGD-containing cyclic heptapeptide	Clinically used	√	√	√	√
**RUC-4**	Low-molecular weight molecule	Used in pre-clinical studies	√	√	X	Not evaluated
**scFv MA2**	Single-chain antibody directed against the activated form of αIIbβ3	Used in pre-clinical studies	√	√	X	No impact on mouse tail-bleeding time
**mAb anti-PSI**	Monoclonal antibody against the β3 PSI domain	Used in pre-clinical studies	√	√	X	No impact on mouse tail-bleeding time
